# Trends in polytrauma incidence among major trauma admissions

**DOI:** 10.1007/s00068-022-02200-w

**Published:** 2022-12-19

**Authors:** Benjamin Maurice Hardy, Kate Louise King, Natalie Enninghorst, Zsolt Janos Balogh

**Affiliations:** 1 Department of Traumatology, John Hunter Hospital, Newcastle, NSW 2310 Australia; 2 University of Newcastle, Newcastle, NSW Australia

**Keywords:** Polytrauma, Trauma, Multiple trauma, Trauma center, Trauma care, Trauma surgery, Background

## Abstract

**Purpose:**

Polytrauma is increasingly recognized as a disease beyond anatomical injuries. Due to population growth, centralization, and slow uptake of preventive measures, major trauma presentations in most trauma systems show a slow but steady increase. The proportional contribution of polytrauma patients to this increase is unknown.

**Methods:**

A 13-year retrospective analysis ending 31/12/2021 of all major trauma admissions (ISS > 15) to a level-1 trauma center were included. Polytrauma was classified using the Newcastle definition. Linear regression analysis was used to compare the rates of patient presentation over time. Logistic regression was used to measure for change in proportion of polytrauma. Data are presented as median (IQR), with odds ratios and 95% confidence intervals as appropriate.

**Results:**

5897 (age: 49 ± 43 years, sex: 71.3% male, ISS: 20 ± 9, mortality: 10.7%) major trauma presentations were included, 1,616 (27%) were polytrauma (age: 45 ± 37 years, 72.0% male, ISS: 29 ± 14, mortality: 12.7%). Major trauma presentations increased significantly over the study period (+ 8 patients per year (3–14), *p* < 0.01), aged significantly (0.42 years/year (0.25–0.59, *p* < 0.001). The number of polytrauma presentations per year did not change significantly (+ 1 patients/year (– 1 to 4, *p* > 0.2). Overall unadjusted mortality did not change (OR 0.99 (0.97–1.02). Polytrauma mortality fell significantly (OR 0.96 (0.92–0.99)) over the study period.

**Conclusions:**

Polytrauma patients represent about 25% of the major trauma admissions, with higher injury severity, static incidence and higher but improving mortality in comparison to all major trauma patients. Separate reporting and focused research on this group are warranted as monitoring the entire major trauma cohort does not identify these specifics of this high acuity subgroup.

The population of all trauma patients admitted to an institution tends to be skewed toward minor injury. For practical reasons, researchers and quality improvement systems use a threshold of anatomical injury severity to specify a cutoff above which is classified as major trauma for analysis. Injury Severity Score (ISS) > 15 as the cutoff for ‘major trauma’ is the most routinely used internationally [1].

Despite injury prevention strategies, and due to an increasing population and centralization of trauma care, the number of major trauma patients continues to increase in most trauma centers [2]. With an increasingly broad group, smaller subgroups of patients may be obscured within the larger patient population, particularly if those groups are not increasing at the same rate.

Polytrauma is recognized as a disease beyond pure anatomical injuries but representing a patient at risk of complications and physiological deterioration [3]. Polytrauma patients have increased risk of systemic inflammatory response syndrome (SIRS) and multiple organ failure (MOF) [4, 5]. They require specialist-led care to manage the competing priorities of different body systems. They consume considerable healthcare resources which may overwhelm less coordinated systems [6]. The quality treatment of polytrauma patients is central to major trauma care. However, due to the predominance of single-system injuries, polytrauma patients are statistically a minority of the major trauma population whose effect size may be crowded out. It is not known whether this proportion is changing over time.

We hypothesized that polytrauma patients were a decreasing proportion of the major trauma population.

## Methods

### Patients

The John Hunter Hospital is a level-1 trauma center located in New South Wales, Australia. It is the highest volume trauma center in the state. It cared for 513 ISS > 15 major trauma patients in 2019 [2]. Since 2002, all major trauma patients have been collected in a trauma registry, and since 2009, granular AIS data have been stored. Patients are included in the registry if they had an Injury Severity Score (ISS) > 15, or admitted to an Intensive Care Unit (irrespective of ISS) following injury; or died in hospital (irrespective of ISS) following injury, except those with an isolated fractured neck of femur injury sustained from a fall from a standing height (< 1 m) and those aged 65 years or older who die with minor soft tissue injury only [2]. In this study, all patients with an ISS > 15 were included. There were no exclusion criteria.

Polytrauma was defined using the Newcastle definition of abbreviated injury score of 3 or more in at least two body regions [4]. Injury severity score was calculated by the sum of the squares of the AIS severity scores of the three most severely injured body regions [7]. Monotrauma was defined as the remainder of the patients in the ISS > 15 group that did not satisfy the polytrauma definition. The most severely injured monotrauma patients have an ISS of 33 (AIS 5^2^ + 2^2^ + 2^2^).

Data were extracted from the prospectively maintained trauma database for the entire period between January 1st, 2009 and December 31st, 2021. Data extracted were age, sex, mechanism, date of injury, ISS, ISS body region and matching AIS score, and in-hospital mortality. Ethical approval was granted by the Hunter New England Human Research Ethics Committee with reference AU202206-13. Data was not deposited in a public registry due to preserve the privacy of human subjects. We used the Strengthening the Reporting of Observational Studies in Epidemiology (STROBE) checklist in writing this manuscript [8].

### Analysis

Data were analyzed using Stata 17 (StataCorp. 2021. Stata Statistical Software: Release 17. College Station, TX: StataCorp LP.). Continuous data were presented as median (IQR), categorical data as counts, proportions and confidence intervals. Summary statistics are presented as either representing the entire ISS > 15 group (ISS > 15), the polytrauma subgroup, or the monotrauma subgroup (polytrauma + monotrauma = ISS > 15). Comparisons between medians were by Kruskal–Wallis Test. Comparisons between proportions was with the Fischer’s Exact test. The effect of the passage of time against number of patients, age and injury severity was tested using single and multiple linear regression and presented as coefficients and confidence intervals. Change in mortality over time was tested using logistic regression and presented as odds ratios and confidence intervals. Statistical significance was set at 5%.

## Results

**Fig. 1:**
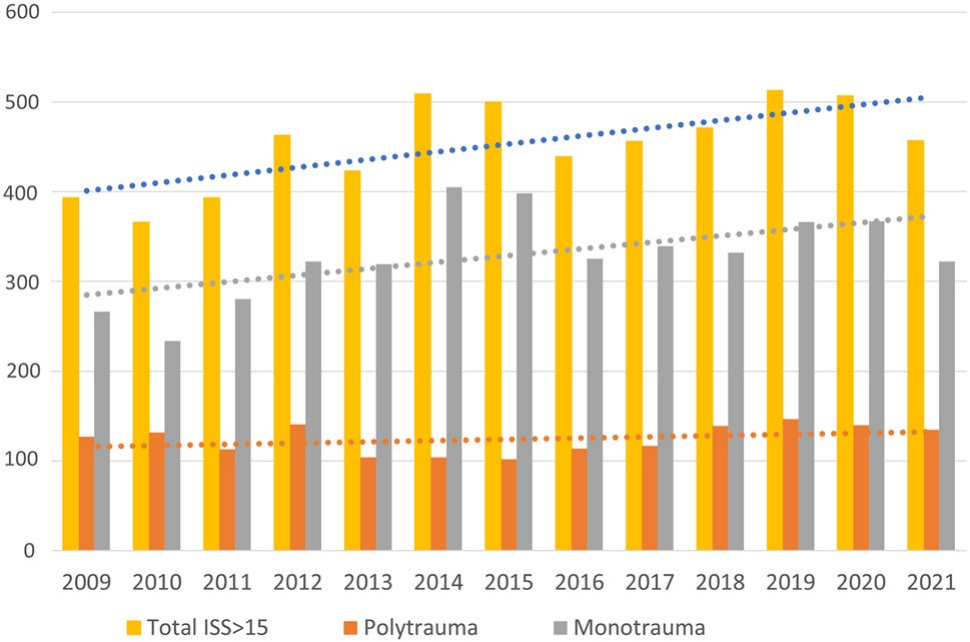
Total major trauma presentations compared with polytrauma presentations

**Fig. 2:**
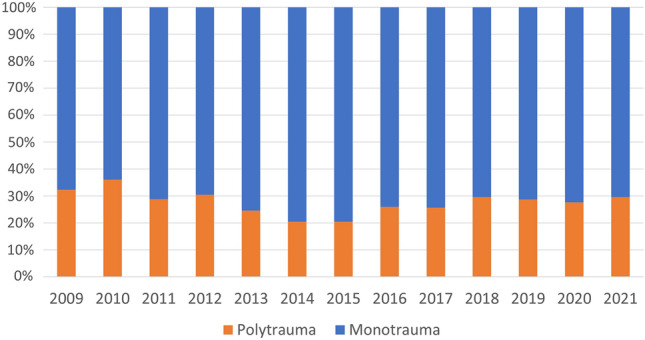
Proportional contribution of polytrauma patients to total major trauma volume

**Fig. 3:**
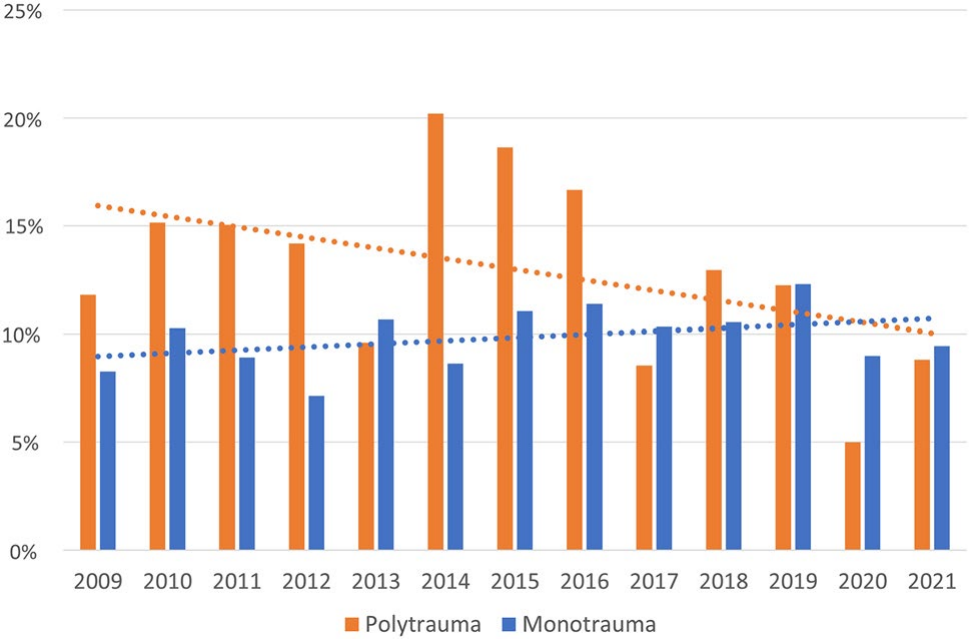
Comparison between annual mortality of polytrauma and monotrauma patients

There were 5,897 ISS > 15 trauma patients between January 1st, and December 31st 2021 contained with the registry. Data were 100% complete for ISS, AIS, and mortality. The number of major trauma patients increased year-on-year (Tables 1 and 2; Fig. 1). Median ISS was 20 (17–26) and did not change over the period (*p* = 0.44). 1616 patients (27.4%) were classified as polytrauma (Fig. 2). Polytrauma patients had a median ISS of 29 (22–36) and this did not vary significantly over the study period (*p* = 0.21). The contribution of head injury to ISS did not change over time (SDC1). There was no difference between the groups in age, sex, or mechanism (Table 1). Age increased over the study period in both the groups (All: 0.42 years/year (0.25–0.59, *p* = < 0.001), polytrauma: 0.53 years/year (0.24–0.81, *p* < 0.001). Overall ISS > 15 in-hospital mortality was 10.6% (9.9–11.5%) and did not change significantly over the study period (Table 2). In-hospital polytrauma mortality was 12.7% (11.1–14.4%) and fell over the study period (*p* = 0.02, Table 2; Fig. 3). Monotrauma mortality did not fall over the study period (*p* = 0.24, Table 2; Fig. 3). Polytrauma mortality was significantly higher than monotrauma mortality (Table 1). The number of polytrauma patients per year did not increase significantly, whereas monotrauma patient presentations increased significantly (Tables 2, 3 and 4; Fig. 1).

**Table 1:** Demographics. #test is between polytrauma and monotrauma *test is between ISS > 15 and polytrauma

	ISS > 15	Monotrauma	Polytrauma	*p*-value#
Number	5897	4281	1616	
Age (yr, IQR)	49 (28–71)	52 (29–74)	45 (25–62)	> 0.2#
ISS (median, IQR)	20 (17–26)	17 (17–25)	29 (22–36)	
*Sex* (*n*, %)				> 0.2#
Male	4,203 (71.3%)	3,039 (71.0%)	1,164 (72.0%)	
Female	1,694 (28.7%)	1,242 (29.0%)	452 (28.0%)	
Penetrating + (*n*, %)	201 (3.5%)	150 (3.6%)	51 (3.1%)	> 0.2#
Mortality (*n*, %)	629 (10.7%)	423 (9.9%)	206 (12.7%)	0.02*

+ Denominator differences represent missing data

**Table 2:** Linear regression of number of patients per year, *p < 0.05 **p < 0.01

	ISS > 15	Polytrauma
Year	8.96 (3.21–14.71)*	1.40 ( – 1.16to 3.95)
Constant	399.85 (359.19–440.50)*	– 2,687.91 ( – 7,830.53 to 2,454.71)

**Table 3:** Logistic regression of mortality, *p < 0.05 **p < 0.01

	ISS > 15	Monotrauma	Polytrauma
Year	0.994 (0.972–1.017)	1.017 (0.989–1.046)	0.956 (0.920–0.993)*
Constant	0.124 (0.105– 0.145)**	0.098 (0.080–0.121)**	0.19 (0.146–0.25)**

**Table 4:** Multiple linear regression of number of patients per year, *p < 0.05 **p < 0.01

Year	*4.94* (4.69–5.19)**
Category	
Monotrauma	0
Polytrauma	– 208.76 ( – 210.80 to – 206.73)**
Constant	– 9,620.70 ( – 10,119.78 to – 9,121.61)**

## Discussion

In our prospective database, polytrauma patients were a falling proportion of major trauma patients. Despite increasing age, mortality has fallen among polytrauma patients. This improvement was not seen in monotrauma group. The reason for this is unknown. During the study period, the trauma surgical service continued to expand and improve. The mortality improvement was also not seen in the overall ISS > 15 population having been obscured by the larger number of monotrauma patients.

By its nature, the major trauma population is a heterogenous group. Single-system disease continues to undergo improvements in outcome including in the elderly [9] and the head-injured [10], but the system and resuscitation improvements that effect polytrauma patients are different [11]. The number and outcomes of polytrauma patients are not routinely reported in the literature or state or national registries [12]. This study is the first to track changes in the proportion of patients experiencing polytrauma over time. Polytrauma are a smaller proportion of major trauma than in our previous series [5] and those reported internationally [13]. Due to the lack of long-term comparative studies, it is not possible to comment if this is just a local feature or a possible global trend in the epidemiology of trauma. Our methodology is easily reproducible and would allow large-scale international assessment, which could be useful for planning purposes in resourcing, training and research.

The definition of polytrauma continues to be debated between exclusively anatomic ‘inclusive’ definitions [4], and physiologically ‘exclusive’ subsets of the anatomic group with increasing complexity [14]. These definitions are different with one being a narrow subset of the other. We have reconfirmed that the Newcastle polytrauma definitely describes a multiply injured population at significantly higher risk of death than the major trauma population, in contrast to other studies [13].

The study is limited by its single-center database nature. Only in-hospital death was captured. Improvements in prehospital care over the study period may have resulted in previously prehospital deaths being included as in-hospital due to improvements in prehospital care and reduction in transport times [15].

Polytrauma patients could represent a fixed number and thus a shrinking proportion of increasing major trauma patients. The outcomes of polytrauma patients change independently of monotrauma and may be obscured in the larger major trauma population. Polytrauma performance metrics should be standardized and reported separately.
